# Safety of various parameter sets with navigated microsecond pulsing laser in central serous chorioretinopathy

**DOI:** 10.1186/s40942-021-00335-3

**Published:** 2021-10-16

**Authors:** Jay Chhablani, Gagan Kalra, Lubna Alkwatli, Bernd Fassbender, Francesca Amoroso, Khushboo Chandra, Samantha Ankireddy, Dmitrii Maltsev, Nina-Antonia Striebe, Eric Souied

**Affiliations:** 1 LV Prasad Eye Institute, Hyderabad, India; 2 University of Pittsburgh, UPMC Eye Center, Pittsburgh, PA 15213 USA; 3 Cole Eye Institute, Cleveland Clinic, Cleveland, OH 44195 USA; 4 Giers, V. Lovenberg-Prömper, Fassbender, Detmold, Germany; 5 Centre Hospitalier Intercommunal de Creteil, Paris, France; 6 School of Medicine, University of Missouri-Kansas City, Kansas, MI USA; 7 Department of Ophthalmology, Military Medical Academy, St. Petersburg, Russia; 8 University Clinic, Göttingen, Germany

**Keywords:** Navigated microsecond pulsing laser, CSCR, Subthreshold, Safety

## Abstract

**Background:**

Subthreshold microsecond pulsing laser is an increasingly common treatment approach for central serous chorioretinopathy. However, there is no literature available on the safety of microsecond laser using different fluence settings in this disease. While many publications can be obtained from conventional microsecond pulsing lasers, few parameter sets are published with the navigated microsecond pulsing laser. Therefore, this study aims to investigate the safety of different parameter sets in subthreshold microsecond pulsing laser treatments.

**Methods:**

In this retrospective chart review, consecutive patients with central serous chorioretinopathy (> 3 months duration of symptoms) treated with navigated subthreshold microsecond pulsing laser and a follow up of at least five months after microsecond laser application were included. For each patient, the treatment parameters, plan layout, and adverse events related to laser were evaluated. Secondary outcomes included best-corrected visual acuity and anatomical improvements (central retinal thickness).

**Results:**

One hundred and one eyes were included in the observation and followed for a mean of 10 months (range 5–36). Although a larger range of parameter sets and fluence settings have been used, no patient demonstrated adverse events from navigated microsecond pulsing laser. While 88% of the cases demonstrated stability, 13 cases lost five or more letters due to the persistence of the subretinal fluid. In mean, a best-corrected visual acuity improvement of 0.07logMar (± 0.2) was seen (p = 0.02). In 51% of the patients, a statistically significant improvement of the central retinal thickness was noted at the last follow-up with a mean thickness reduction of 70 µm (± 143) (p < 0.01).

**Conclusion:**

In conclusion, none of the used parameter sets lead to tissue damage (when using a cautious titration) and, in summary, lead to an improvement in subretinal fluid and improvement in visual acuity. However, further prospective studies are needed to correctly identify the dependency of the treatment strategy on the outcome criteria.

## Introduction

Over several years, focal laser photocoagulation has been established as an option to treat central serous chorioretinopathy (CSCR) for extrafoveal lesions. However, side effects such as scarring, choroidal neovascularization (CNV), damage to photoreceptors lead to the reduction of visual benefits to the patients [1]. In the past few decades, more in the last decade, microsecond pulsing laser has evolved to treat retinal disorders. In this type of laser, the continuous wave (CW) laser is split into microsecond pulses to provide a therapeutic effect without causing any damage to the retinal tissue but causing the expression of heat shock protein [2]. Another option for chronic CSCR is photodynamic therapy, which is considered superior to microsecond pulse laser in terms of proportion of patients with complete resolution of subretinal fluid; however, being an invasive procedure and has a risk of overdosing and collateral damage retinal damage [3, 4].

In CW lasers, the endpoint of visible greyish-white color leads to structural changes, which can be seen in imaging studies. On the contrary, there are no detectable changes or damage seen clinically as well as on imaging studies using microsecond pulsing lasers [5, 6]. While microsecond pulsing lasers avoid thermal damage to any layer, it remains effective by inducing the expression of heat shock proteins [2] as well as protein and mRNA expressions of angiogenic proteins (VEGF-A, TGF-β, and bFGF), that were significantly down-regulated (P < 0.05), whereas those of the angiogenic inhibitor (PEDF) were up-regulated (P < 0.05) [7]].

There are different types of microsecond pulse lasers commercially available. The first differentiation factor is the wavelength used, either 810 nm (infrared) or 577 nm (yellow). While the 577 nm yellow wavelength maintains a negligible uptake by macular xanthophyll and has the highest absorption rate for oxyhemoglobin to melanin absorption ratio, the 810 nm lasers have a very limited scattering in media opacities but present with relatively poor absorption by the retinal pigment epithelium (RPE) and choroid which requires higher power [8]. Although the yellow wavelength laser seems to be more popular in recent times [9, 10] with less power required [11] and potentially less pain when using the laser for threshold applications [12], it remains debated which wavelength is optimal for the microsecond pulsing laser application. For example, Chang et al. found 810 and 577 nm to be equally effective, but 810 nm had a significantly wider therapeutic range/safety margin [13]. The authors state that the small therapeutic range of microsecond pulsed leads to less safety and a higher incidence of inadvertent laser burns. Such valid criticisms show the imminent need for detailed analysis of the safety of yellow wavelength laser devices.

Since 2015 multiple retrospective and prospective case series and smaller randomized controlled trials exist for the treatment of CSCR with yellow wavelength microsecond pulsing laser treatment that shows a consistent positive effect on the resolution of subretinal fluid in CSCR. Success rates vary from ~ 40% at 3 month [14] and 70% at 6 months [15] up to 92% at 12 months [16]. While the results are relatively consistently positive in these papers, there is a larger disagreement on the optimal parameter set, consisting of duty cycle, spot size, pulse duration, used titration method, and side effects of these variable parameter sets. There also is a larger controversy if a fixed parameter set or a titrated initiation is the optimal method to use. While Luttrull defends using a fixed parameter set for any indication, especially with 810 nm subthreshold lasers [13], there is less consistency in the parameter selection for yellow wavelengths lasers. Donati et al. compared a fixed and a titrated approach for treating diabetic macula edema using a yellow wavelength laser and found both approaches similarly effective but did not specifically analyze the safety of each parameter set[17]. In CSCR treatment publications, fixed-parameter sets have been used as well with 5%DC, 200 ms, and a power value between 250 and 400mW [18–22]. Most of the published literature on CSCR used titrated approaches, although the titration methods vary widely as well but generally rely on titrating in microsecond pulsing mode and then reducing the power to 30% or 50% of the level of a barely visible burn [14, 23, 24]. The threshold of 30% or 50% of the level of a barely visible burn had been based on the work by Palanker et al. In one study, Wang and Palanker analyzed the thermal damage and the level of heat shock protein expressions in an animal model for a certain range of parameters and defined 30% of the threshold power to be safe [5]. However, the safety of the 30% threshold has not been analyzed for most of the generally used parameter sets (such as 100 µm, 100 ms) and has not been analyzed in a real-world situation [5]. The present study aims to evaluate the safety and adverse events of different microsecond pulsing parameter sets in patients with central serous chorioretinopathy in a real-world setting.

## Methods

This is a retrospective chart review of consecutive patients from five sites (India, Germany, France, Russia). Patient informed consent had been obtained from all patients. The study was approved by the respective local ethics committee and adhered to the tenets set forth in the Declaration of Helsinki.

Patients with chronic or recurrent leakage in central serous chorioretinopathy (symptoms > 3 months) treated with navigated microsecond pulsing laser (Navilas® Laser System) and at least five months follow up after laser treatment were included. Each site selected the settings individually. The basic principle for the parameter set selected was defined per literature review individually at each site when they started introducing microsecond pulsing laser treatments. In the following months/years, each site modified the parameters based on their clinical and more practical aspects, such as the size of diffuse edema (e.g., increasing spot size) and location on the retina (e.g., modifying pulse duration).

Exclusion criteria included patients with a history of other laser treatments, including photodynamic therapy; conventional laser; patients with any other coexistent ocular disease. As it is the standard of care in certain sites to first treat with oral treatment such as mineralocorticoid antagonists, we included such cases because they do not impact the safety of a microsecond pulsing laser.

The primary objectives of this study were the incidence of adverse events related to laser applications in correlation to the fluence and parameter settings. Adverse events were defined as presence of laser-related durable tissue defect (detectable in OCT) and as the development of CNV, atrophic scars, or similar. A vision loss of more than five letters (one line on Snellen's chart or 0.1logMAR) related to laser treatment was evaluated as a serious adverse event. The secondary objectives included the percentage of re-treatment with any additional intervention (including microsecond pulsing lasers) and the resolution rate of subretinal fluid (partial, complete, persistent, worsened, and recurrent). Complete resolution was defined as the complete disappearance of the subretinal fluid (SRF). Partial resolution, if the SRF was reduced significantly (more than 20% thickness reduction). Persistent, when the SRF height did not significantly change (less than 20% change, or even increase >  + 5% change in highest elevation). Recurrent SRF was counted as partially resolved.

All data were collected in an excel sheet and evaluated using RStudio (RStudio inc., USA). Independent variables included all laser parameters, as well as the titration method. Descriptive analysis was used with providing mean, standard deviation, and range. Test for differences was done using Wilcoxon signed-rank test for best-corrected visual acuity and thickness measurements. A P-value of < 0.05 was considered statistically significant.

## Results

A total of 101 eyes of 86 patients with CSCR who underwent navigated microsecond laser with previously specified inclusion criteria were included in the study. The patient baseline characteristics are outlined in Table 1. The mean age of the patient was 50, with a range from 24 to 76, with the majority of patients being male (73%). Fifty percent of the patients had a chronic, non-resolving type of CSCR. The baseline best-corrected visual acuity of 0.35 ± 0.3 logMar and a baseline central retinal thickness of 325 ± 130 µm. Twenty-seven eyes had been treated with eplerenone (25 mg daily) before the laser treatment. The medication was discontinued at the moment of subthreshold laser treatment. The mean follow-up time of the patients was ten months with a range of 5 to 36.

**Table 1:** Outline of baseline characteristics for complete cohort

	All patients
# of eyes	101
Age (median, range)	50 (24–76)
Follow up time in months (mean–range)	10 (5–36)
Gender	73% male
	27% female
% of patient with systemic medication (eplerenone/spironolactone, 25 mg daily)	26%
Diagnosis	
Acute CSCR	3 (3%)
Chronic	50 (50%)
Persistent	31 (30%)
Recurrent	17 (17%)
BCVA in logMar (mean, SD)	0.35 ± 0.3
CRT in µm (mean, SD)	325 ± 130
Type of leakage	
Diffuse	58 (57%)
Focal	43 (43%)

*CRT* central retinal thickness, *SD* standard deviation

Several different parameter sets have been used and thus influencing the final fluence applied per spot with a mean applied fluence of 206 ± 171 mJ/mm^2^, with a range of 19 mJ/mm^2^ to 881 mJ/mm^2^ (see Table 2). The applied fluence per spot is significantly different between the parameter sets (p < 0.005).

**Table 2:** Characteristics of Parameter sets used

	Parameter Set 1	Parameter Set 2	Parameter Set 3	Parameter Set 4	Parameter Set 5	Parameter Set 6	Parameter Set 7	Parameter Set 8
# of eyes	12	43	9	27	4	1	4	1
Duty Cycle	5%	5%	5%	5%	15%	2.5%	10%	5%
Spot Size (in µm)	100	200	100	100	200	100	100	100
Pulse Duration (in ms)	200	200	100	100	200	100	100	100
Titration / Power definition method	MSP, 30%	MSP, 30%	MSP, 30%	MSP, 70%	MSP 30%	MSP 30%	CW, doubled power	CW, doubled power
Area of treatment	area over the leak for focal, for diffuse, area of SRF							
Power (mW)	196 ± 124	222 ± 95	340 ± 113	662 ± 204	113 ± 10	575 (na)	125 ± 25	140 (na)
fluence per spot (mJ/mm^2^)	250 ± 157	71 ± 30	216 ± 72	422 ± 130	107 ± 9	146 (na)	159 ± 32	89 (na)
# of spots	78 ± 141	220 ± 232	169 ± 147	97 ± 115	438 ± 263	33 (na)	276 ± 423	207 (na)

Values for power, fluence and number of spots given in Mean and Standard Deviation

*MSP* Microsecond pulse mode, *CW* continuous wave, *SRF*subretinal fluid

### Safety outcomes

None of the cases included in this retrospective evaluation experienced any adverse event from microsecond laser on clinical examination and imaging studies at the final follow-up. Twenty-seven (27%) eyes underwent more than a single session of microsecond laser during the follow-up period, and none showed any signs of laser-induced damage.

### Visual outcomes

**Fig. 1:**
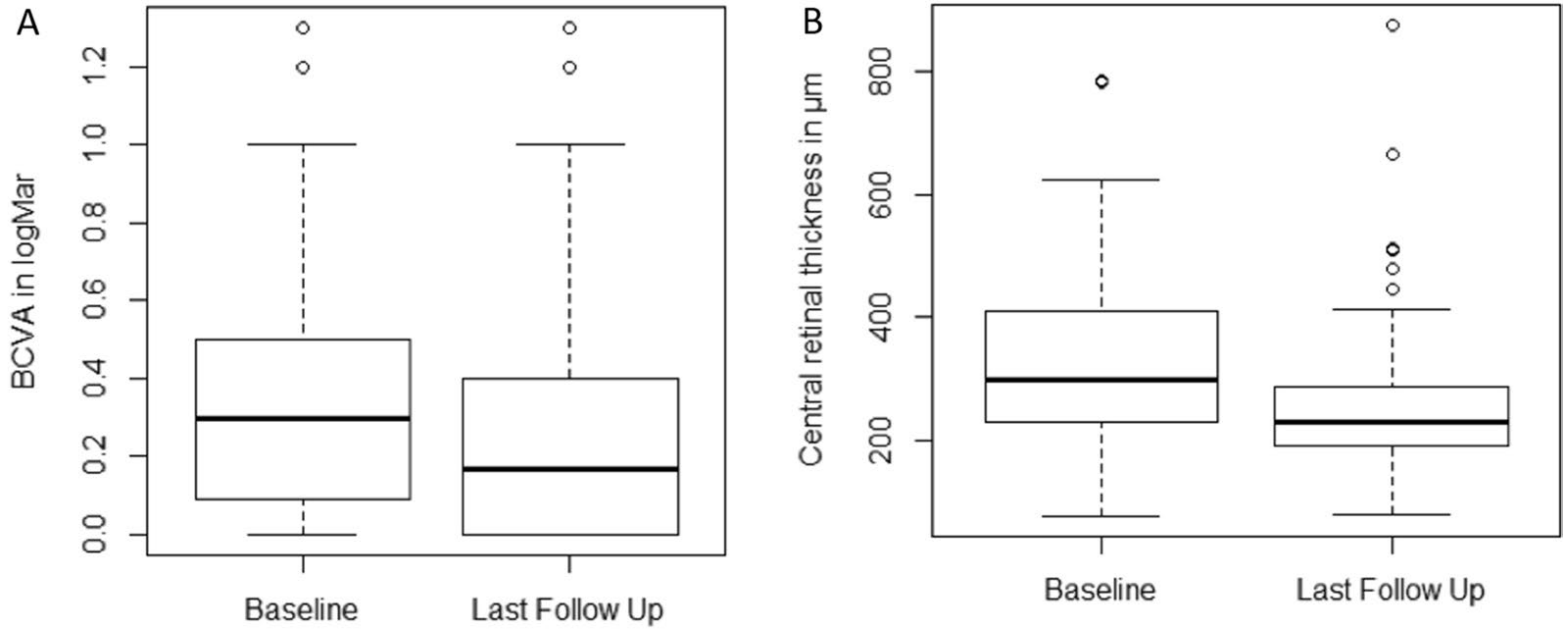
Visual Outcomes of the complete cohort measured in Best Corrected Visual Acuity (BVCA) and Anatomical outcomes of the complete cohort measured in Central Retinal Thickness (in um)

A total of 88% of the cases remained stable or demonstrated improvement in visual acuity. The visual acuity improved by 0.07 logMar, as seen in Fig. 1. At the same time, 13 cases (13%) lost five letters or more during the follow-up time of in mean ten months (range 5 to 36). No consistency in the characteristics of both patient groups could be identified. All cases with either BCVA loss of at least five letters were reviewed, and, in all cases, a persistent or increasing subfoveal subretinal fluid was the cause for the reduced visual acuity. None of these cases show any signs of laser-induced damage.

### Anatomical outcomes

The resolution rate and visual acuity improvements for all patients are outlined in Table 3. During the follow-up period of 10 months (range 5 to 36), 68% of the patients showed an improvement of the subretinal fluid, while the remaining 30% showed a persisting subretinal fluid and 2 (2%) cases demonstrated a worsened subretinal fluid. Among the eyes with improvements of subretinal fluid, 51% of the cases demonstrated a complete resolution of the subretinal fluid, 12% a partial resolution, and 2% completely resolved but recurred within the follow-up time.

**Table 3:** Result overview of the complete cohort values given in mean and standard deviation

	All patients
Total Number of included eyes	101
Resolution of subretinal fluid	
Worsened	2 (2%)
Persistent	31 (31%)
Recurrent	2 (2%)
Partially resolved	12 (12%)
Completely resolved	54 (54%)
BCVA development	
Baseline	0.35 ± 0.3
Last follow up	0.27 ± 0.31
P	0.02*
Gain	− 0.07 ± 0.2
Patients with > 5 letters loss	13 (13%)
Patients with stability	53 (53%)
Patients with > 5 letter gain	35 (35%)
CRT reduction	
Baseline	325 ± 130
Last follow up	255 ± 115
P	< 0.001*
Loss	− 70 ± 143
Number of MSP treatment	1.34 (1–3)
Additional treatment with other treatment modality	9/101 (9%)
adverse events from laser	0/101 (0%)

Values given in mean and Standard deviation

*CRT* Central retinal thickness, *MSP* microsecond pulsing laser, *BCVA* Best Corrected visual acuity

A complete resolution has been achieved in 51 cases by microsecond pulsing laser alone (51% of the whole cohort, 94% of the patient with complete resolution cohort). In 9% of the cases (whole cohort), the treating physician decided to use an additional intervention with either eplerenone (2 cases), spironolactone (1), PDT (2), focal laser (2), or intravitreal injection (2) to achieve optimal results. However, this did only lead in 3 cases to a complete resolution of subretinal fluid.

## Discussion

The present study demonstrates no detectable structural damage on clinical examination and imaging studies following microsecond pulsing lasers in eyes with CSCR. More importantly, this study establishes the safety of microsecond lasers across a variety of different parameter settings. Approximately 2/3rd of the patients showed an improvement in the anatomical presentation. A statistically significant improvement was found for BCVA and CRT in the study cohort.

While evaluating subthreshold laser using CW laser, Lavinsky et al. 2014 found that lesions treated with 50% to 75% energy level were typically subvisible ophthalmoscopically[25]. However, the lesions were detectable with fluorescein angiography and optical coherence tomography. Histology of these lesions demonstrated some selective damage to retinal pigment epithelium and photoreceptors. Although a certain number of cases in our study were treated at the level of 70% of the threshold, no patient showed any long-lasting damage. However, Lavinsky et al. only analyzed this using a continuous wave laser instead of a microsecond pulse laser (MSP) [25]. Wang et al. compared CW to MSP 5% Duty Cycle; 200 ms and noted a damage threshold of 60% of the immediately visible power threshold [5]. Still, this value is below the 70% setting in several of these patients. Both studies (Lavinsky et al. and Wang et al.) analyzed these settings in healthy retinas of animal models [5, 25]. Although care should be taken not to compare animal models with a human application directly, this gives a first indicator of the safety of a specific parameter set. Our clinical study also showed no structural damage in eyes treated with CSCR with 30% and 70% threshold. Two measures might have ensured this absence of structural damage. First, a highly cautious titration procedure was performed. Secondly, placement of all subthreshold burns was limited to affected structures only, rather than non-elevated areas.

Scholz et al. (2017) provide an overview of studies using microsecond pulsing lasers used for central serous chorioretinopathy [26]. In this overview, out of 276 eyes treated with microsecond pulsing laser included from 18 studies, one case report resulted in vision reducing tissue defects27, while only 1 study was noted to have RPE changes at follow-up without complications [28]. In the study of Lanzetta et al., an 810 nm laser had been used at 15%DC and 200 ms Pulse durations and a mean of 1350mW [28], while the case report by Gawecki et al. used 577 nm, at 160 µm, 5% DC, 0.2 s, power: 550 mW (50% of threshold) [27].

A favorable anatomical outcome using microsecond laser in CSCR having been varying from 10 to 86% percent with various parameters and different lasers. Scholz et al. listed a study with a complete resolution rate of subretinal fluid between as low as 10% (Breukink et al., with 810 nm and no high-density grid) [29] up to as high as 86% in Elhamid using a 577 nm, 200 µm, 10% DC, 0.2 s, power tripled as compared to the CW threshold [30]. Our study had results with 54% of complete resolution with a 577 nm microsecond laser.

Except for the Navilas® laser system, all microsecond pulsing lasers use the slit lamp as the delivery method with manual positioning and handwritten documentation. The Navilas® uses a digital imaging concept that allows pre-planning of the microsecond pulsing laser spots, followed by computer-assisted application of the single laser spots. Thus, reliable confluency over large areas and accurate documentation of the treatment are provided. Therefore, navigated microsecond pulsing laser was selected to be the laser for this study to ensure the similarity of the treatment strategies of all patients as the treated locations can be documented and exported. In addition, Navilas limits the risk of overlapping spots or doubled areas due to its ability to track the laser and the eye.

The study's strength is a relatively large sample size with a broad range of generally used laser parameters. Also, the elimination of the inter- and even intra-operator variability is minimized due to the use of a navigated planning concept.

One of the major limitations of the study is the retrospective evaluation without a control arm. Different from other publications, where fluorescence angiography in addition to OCT imaging is used to define tissue defects, our study relied on optical coherence tomography analysis of the retinal structure to identify potential changes. This is mainly caused by the fact that not all patients required another fluorescein angiography in the clinical routine. However, high-quality optical coherence tomography is sensitive enough to detect changes caused by subthreshold laser [31]. An additional limitation is the use of oral mineralocorticoid antagonists as pre-treatment in 26% of eyes which may have affected the anatomical and visual outcome. In addition, the study is not powered enough and not designed to identify differences in the outcomes by parameter set. Additional studies or an extension of the present data set seem to be required to identify the optimal parameter set. All our treatments had been performed using a yellow laser wavelength and navigation system. Therefore, our results cannot be extrapolated to other lasers with different wavelengths.

In conclusion, none of the used parameter sets lead to tissue damage (when using a cautious titration) and, in summary, lead to an improvement in subretinal fluid and improvement in visual acuity. However, further prospective studies are needed to correctly identify the dependency of the treatment strategy on the outcome criteria.

## Data Availability

The data can be sought upon request from the corresponding author with appropriate data sharing agreements in place.
